# Dietary iron attenuates *Clostridioides difficile* infection via modulation of intestinal immune response and gut microbiota

**DOI:** 10.1080/21505594.2025.2529454

**Published:** 2025-07-16

**Authors:** Xiao Li, Xiaoxiao Wu, Wanqing Zang, Zhou Zhou, Wenwen Cui, Ying Chen, Huan Yang

**Affiliations:** a Xuzhou Key Laboratory of Laboratory Diagnostics, School of Medical Technology, Xuzhou Medical University, Xuzhou, Jiangsu, China; b Microbiology Laboratory, Xuzhou Center for Disease Control and Prevention, Xuzhou, Jiangsu, China

**Keywords:** Dietary iron, *Clostridioides difficile*, CDI, gut microbiota, metabolites

## Abstract

*Clostridioides difficile* (*C. difficile*) is one of the majors causes of antibiotic-associated diarrhea globally. Host vulnerability to *C. difficile* infection (CDI) is largely affected by gut microbiota, which in turn is influenced by diet. However, the mechanism underlying the interplay between diet and the gut microbiota that regulates host susceptibility to CDI remains unclear. This study aimed to investigate how a high-iron diet affects the intestinal immune response, microbiota, and metabolism in mice infected with *C. difficile*. We explored the specific role of the unique gut microbiota and metabolites on CDI. A mouse model of CDI was constructed with or without high dietary iron treatment. The effect of high iron levels on gut microbiota was analyzed by 16S rRNA gene sequencing, and the role of gut microbiota was confirmed by fecal microbiota transplantation (FMT). High dietary iron (400 mg/kg ferrous sulfate) alleviated CDI by decreasing *C. difficile* pathogenicity and altering host intestinal neutrophil recruitment. Furthermore, *E. coli AVS0501*, enriched in the gut microbiota of iron-treated CDI mice, showed prophylactic and therapeutic effects on CDI. Moreover, the production of L-proline and tauroursodeoxycholic acid (TUDCA) in CDI mice treated with high dietary iron influenced *C. difficile* colonization, toxin production, and in turn, regulates the intestinal neutrophil response. Thus, high dietary iron alleviates *C. difficile* induced enteritis by regulating gut microbiota maintaining gut homeostasis, suggesting that high dietary iron may be an important determinant of disease control.

## Introduction

*C.difficile* is a gram-positive, spore-forming, obligate anaerobic bacillus, which thrives in the intestinal niche [1]. *C. difficile* infection (CDI) stands as a significant healthcare-associated disease, which causes diarrhea, pseudomembranous colitis, and toxic megacolons [2]. CDI is the fifth leading cause of death due to non-malignant gastrointestinal diseases in the USA [3]. Antibiotic administration, a common antecedent to CDI, disrupts the protective symbiosis between the host and gut microbiota, eroding colonization resistance [4]. Broad-spectrum antibiotics deplete commensal bacteria such as *Bacteroidetes* and *Firmicutes*, which normally produce short-chain fatty acids and secondary bile acids that inhibit *C. difficile* growth [5,6]. This dysbiosis creates an ecological niche where *C. difficile spores* can germinate in the colon. Triggered by bile acids like taurocholate and amino acids like glycine, spore germination releases vegetative cells that secrete toxins, driving mucosal inflammation and barrier disruption [7]. Vancomycin or fidaxomicin is used for primary CDI, and FMT can be used for severe CDI [8–10]. However, even with these treatments, one in nine patients over 65 years old will die within 30 days of diagnosis [11]. These findings highlight the importance of alternative CDI treatment strategies.

The association between diet and susceptibility to CDI has recently become an important research topic, as nutrient regulation affects susceptibility to infection [12–14]. In addition to major dietary components, micronutrients also have a profound effect on innate immunity and susceptibility to infection [15,16]. Dietary metals have been linked to susceptibility to numerous infections, specifically, iron is an essential micronutrient that contributes to various physiological processes [17–19]. Moreover, iron is a vital micronutrient for bacterial metabolism, plays a dual role in bacterial pathogenesis. Bacteria require iron for enzymatic reactions, yet excess iron induces oxidative stress through reactive oxygen species (ROS) generation [20,21]. Cernat *et al* found that *C. difficile* has multiple iron-uptake systems that facilitate adaptation to both iron-overloaded and iron-restricted environments [22]. A recent discovery highlights the role of “ferrosome,” the membrane-bound iron storage organelles within *C. difficile*, formed under iron-limited conditions, to combat iron sequestration by the host in the inflamed gut during infection, thereby enhancing its survival and virulence [23–25]. Importantly, Skaar and colleagues discovered that patients with CDI have significantly lower levels of Fe than the control group [23]. However, the association between host iron levels and infection is complex, and the role of high iron levels in CDI remains unclear.

Diet is an important regulator of microbial communities, and dysbiosis of gut microbiota can promote CDI. Some specific nutrient-rich diets, such as high-fat, high-zinc, and high-protein diets, exacerbate CDI by altering the gut microbiota [13–15]. Although specific diets affect susceptibility to CDI, the mechanism underlying dietary iron regulation of the gut microbiota to determine the outcome of CDI remains unclear. A low-iron diet has been linked to prolonged survival in parasitic infection and limited disease pathology in bacterial infections [26]. Therefore, the effect of iron on CDI may depend largely on interactions between host iron levels, immune status, and gut microbiota composition.

Here, we investigate the effects of a high-iron diet on the intestinal immune response, microbiota, and metabolism in mice infected with *C. difficile*. By exploring how iron modulates gut microbial communities and their metabolites, we aim to uncover mechanisms by which dietary iron influences CDI susceptibility. Our data suggest that a high-iron diet may reduce CDI susceptibility by reshaping specific gut microbial populations and metabolites, offering insights into nutrient-based interventions to complement existing therapies.

## Methods

### Mice

Wild-type male C57BL/6J mice, aged 4–6 weeks, were purchased from Xuzhou Medical University, bred and maintained under specific pathogen-free (SPF) condition. All animal experiments were performed according to the standards guide for the Care and Use of Laboratory Animals (Institute of Laboratory Animal Resources of the National Research Council, United States) and ethical approval for the study was granted by Laboratory Animal Ethics Committee of Xuzhou Medical University (IACUC number: 202203A393, Xuzhou, China). All the experimental procedures were performed in accordance with the ARRIVE Guidelines 2.0.

### C. difficile spore preparation

*C.difficile* VPI 10,463 (ATCC 43,255) spores were prepared as described previously [27]. VPI 10,463 strain was grown at 37 °C on Brain Heart Infusion (BHI) agar plates (CM1135, OXOID, England) supplemented with 0.05 % L-cysteine (A600132, Sangon Biotech) in anaerobic atmosphere for 2 days, followed by anaerobic culture in BHI broth containing 0.05 % L-cysteine at 37 °C for 2 days. Then the inoculum was added to fresh medium, incubated at 37 °C for 5 days anaerobic. The cultures were harvested by centrifugation and washed with PBS. The pellets were suspended in PBS and heat treated for 10 min at 70 °C to inactivate vegetative cells and obtain *C. difficile* spores. Viable spores were plated on BHI agar plates supplemented with 0.1 % l-cysteine and 0.1 % sodium taurocholate (A601143, Sangon Biotech). The number of viable spores was recorded as CFU/mL. Spore stocks were stored at −20 °C.

### Mouse model of CDI

The mouse model of primary and recurrent CDI were established as described previously [27], 6 days before infection, mice were pretreated with antibiotic cocktail (ABX), the antibiotic mixture of gentamicin (0.035 mg/mL, A506614, Sangon Biotech), kanamycin (0.4 mg/mL, A506636), metronidazole (0.215 mg/mL, A600633), polymyxin E (0.035 mg/mL, A606495), and vancomycin (0.045 mg/mL, A600983) were added to drinking water to effectively removed the intestinal microbiota of mice. After five days, the mice were intraperitoneally injected with 10 mg/kg clindamycin (A600312). One day later (Day 0), each mouse in the infection group was infected with 5 × 10^6^ CFU of *C. difficile* spores by oral gavage. Body weight and disease symptoms (stool characteristics, weight loss, and decreased response to stimuli) were recorded every 12 h during infection, and mortality was tracked. Disease activity index (DAI) was scored as described previously [28]. The cecal contents, cecum, and colon were harvested 48 hpi. 0.4 mg/mL vancomycin was added to the drinking water for 5 days prior to infection to induce rCDI.

To explore the effects of a high-iron diet on CDI, the mice were randomly divided into four groups. Among these, two groups were fed a normal and high-iron diet for 3 weeks without infection, designated as the NC group (*n* = 6) and NC+Iron diet (*n* = 6) group, respectively. The other two groups were fed a normal and high-iron diet for 3 weeks, followed by infection with *C. difficile* spores, designated as the CDI group (*n* = 6) and CDI+Iron diet group (*n* = 6), respectively. All dietary feed was purchased from XIETONG. ORGANISM, the normal diet contains 100 mg/kg of iron (ferrous sulfate), and the high-iron feed contains 400 mg/kg of iron.

For FMT, 200 mg of stool was collected from the donor mice, resuspended in 1 mL sterile water supplied with 0.05% L-cysteine, and then passed through a 70 μm filter to remove large particulates. The recipient mice were orally administered with 200 μL fresh fecal filtrates for 2 days after infection.

To study the major effect of individual bacterial strains on CDI, mice were gavaged with 1 × 10^9^ CFU of bacterial strains once per day for 10 days before infection.

To investigate the effects of L-proline and TUDCA on CDI, mice in the CDI+proline-free (Pro-free) group were given proline-deficient feed (XIETONG.ORGANISM) for one week before infection. Mice in the CDI+TUDCA group were administered 100 mg/kg TUDCA (Solarbio, China) by gavage for one week before CDI.

### Gross and histological assessment

The entire colon was separated from the end of the cecum to the anus, and was photographed, then the colon length was measured. Isolated cecum was fixed with 4% paraformaldehyde and embedded in paraffin for histological analysis. Tissue sections (4 μm) were stained with hematoxylin and eosin. Images were captured using a microscope (DP74, Olympus). Histological scores were scored based on epithelial tissue damage, edema, and inflammatory cell infiltration. Each category was scored from 0 to 3, with individual values added for an overall score.

### Goblet cell staining

The tissue was fixed in 10% phosphate-buffered formalin for 48 h, then embedded in paraffin and cut into 4 μm sections. AB-PAS staining was performed using an AB-PAS Stain Kit (Servicebio, Wuhan, China) according to the manufacturer’s instructions. Images were captured with an Olympus DP74 microscope. The total number of goblet cells in crypts was counted.

### Immunohistochemistry (IHC)

The tissue was fixed in 10% phosphate-buffered formalin for 48 h, then embedded in paraffin and cut into 5 μm sections. For immunohistochemistry analysis, tissue sections were deparaffinized, dehydrated, and subjected to antigen retrieval in citrate buffer (pH 6.0). The sections were then exposed to 3% hydrogen peroxide, blocked with 5% bovine serum albumin for 30 min, and stained with anti-MUC2 antibody (1:1000, 27675–1-AP, Proteintech) overnight. The sections were washed three times with PBS and stained for 1 h with an HRP-labeled secondary antibody. Staining was developed using a diaminobenzidine chromogenic substrate. Finally, the sections were counterstained with hematoxylin, dehydrated, and mounted. Images were acquired under an Olympus DP74 microscope, and staining intensity was analyzed using ImageJ software.

### ELISA

Lipocalin-2 (LCN2) in feces and cecal contents was quantified using a Mouse LCN2 ELISA KIT (ZCIBIO, China) according to the manufacturer’s instructions. Colons were homogenized in RIPA lysis buffer (KeyGEN, China) containing protease inhibitors (Cwbio, China). Homogenates were centrifuged for 10 min at 10,000×g, and supernatants were collected and used for measurement of IL-1β, IL-6, TNF-α, IL-36 G, and CXCL1. IL-1β, IL-6, and TNF-α levels were measured using a Mouse Uncoated ELISA Kit (Invitrogen, USA), according to the manufacturer’s instructions. IL-36 G was quantified using a Mouse ELISA kit (DLDEVELOP, China). CXCL1 expression was quantified using a Mouse ELISA kit (Fine, China).

### Quantification of *C. difficile*

Mice were sacrificed at 48 hpi, if a mouse died, then it was excluded. The cecum and colon were aseptically removed, weighed, homogenized in 1 mL of PBS containing 0.3% Triton X-100, serially diluted, plated on BHI agar, and the CFU per gram of tissue was counted. Cecal contents and feces were weighed, *C. difficile* genomic DNA was extracted using the TIANamp Bacteria DNA Kit (TIANGEN, China) and mRNA levels were quantified by qPCR using TcdB primers (Table 1). The copy numbers of the contents and feces samples were extrapolated from the standard curve generated with the extracted DNA from known *C. difficile* inocula.

**Table 1.** Primer sequences used in this study.

Primers	Sequence (5’to 3’)
*b-actin*-F	GGCTGTATTCCCCTCCATCG
*b-actin*-R	CCAGTTGGTAACAATGCCATGT
*Tnfα*-F	GATCGGTCCCCAAAGGGATG
*Tnfα*-R	TTTGCTACGACGTGGGCTAC
*Il6*-F	AGACAAAGCCAGAGTCCTTCAG
*Il6*-R	GAGCATTGGAAATTGGGGTAGG
*Il36g*-F	TCCTGACTTTGGGGAGGTTTT
*Il36g*-R	TCACGCTGACTGGGGTTACT
*S100a8*-F	AAATCACCATGCCCTCTACAAG
*S100a8*-R	CCCACTTTTATCACCATCGCAA
*S100a9*-F	ATACTCTAGGAAGGAAGGACACC
*S100a9*-F	TCCATGATGTCATTTATGAGGGC
*Cxcl1*-F	CACCCAAACCGAAGTCATAGC
*Cxcl1*-R	GAAGCCAGCGTTCACCAGA
*TcdB*-F	GGCAAATGTAAGATTTCGTACTCA
*TcdB*-R	TCGACTACAGTATTCTCTGAC

### Quantification of toxins

The levels of *C. difficile* toxin TcdB in feces, cecal contents, and cecum, were determine by a Mouse CDT-B ELISA KIT (Mlbio, China), and samples were processed as recommended by the manufacturers. The samples were normalized according to their weight.

### Lymphocytes preparation

To isolate lymphocytes from the colonic lamina propria, the colon tissues were opened longitudinally, washed with cold PBS, and cut into 1 cm pieces followed by shaking in PBS, then incubated with 10 mM EDTA at 37 °C for 30 min to remove epithelial cells. Then, the tissues were digested with RPMI 1640 medium containing 5% FBS (HY-T1000, ExCell Bio, Uruguay), 1 mg/mL collagenase (11088866001, Sigma-Aldrich, USA), 1×Penicillin-Streptomycin Solution (C0222, Beyotime, China), 1 mg/mL hyaluronic acid (935166, Sigma-Aldrich), and 1 μg/mL DNase I (D806930, MACKLIN) at 37 °C for 1 h. After incubation, the digested solution was filtered through a 70-μm cell strainer to obtain single cell suspensions. Then the cell pellets were resuspended in 40% percoll, followed by centrifugation at 670×g for at 4 °C 30 min to obtain lymphocytes. The cell pellets were washed with PBS and resuspended in PBS containing 2% FBS.

### Flow cytometry

For neutrophil analysis, single cell suspensions were stained for 30 min at 4 °C in the dark with the following antibodies: anti-CD45 conjugated to PE-Cyanine7 (1:200, 147704, I3/2.3, BioLegend, USA), anti-Ly6G conjugated to PE (1:200, 127607, 1A8, BioLegend), and anti-CD11b conjugated to AlexPacific Blue (1:200, 101224, M1/70, BioLegend).

### Neutrophil culture

Firstly, bone marrow was obtained from the long bones of sacrificed mice. Neutrophils were prepared from the bone marrow suspensions through double-gradient centrifugation using Histopaque-1077 and Histopaque-1119 (50/50 v/v; Sigma). Purified neutrophils (1 × 10^5^ cells/mL) were seeded in 6-well plates and cultured at 37 °C in DMEM containing 10% FBS. Initially, the cells were incubated with 50 μM or 500 μM ferrous sulfate for 2 h. Then, *C. difficile* (1 × 10^7^ CFU/mL) was added to the culture for the next 4 h. Subsequently, the culture supernatants were colleted, and IL-1β was quantified by ELISA.

### RNA extraction and qRT-PCR

RNA was extracted from the colonic tissues and converted to cDNA using a PrimerScript RT Reagent Kit (RR037A, Takara, Japan). Then, real-time polymerase chain reaction (PCR) was performed using SYBR Green qPCR Master Mix (b21203, Bimake, USA) on a 7900 Fast Real-Time PCR System (Roche, Switzerland). β-actin was used as an endogenous control. 2^−ΔΔCT^ method was used to calculate the relative gene expression. Primers used are listed in Table 1.

### 16S rRNA gene sequencing

For microbiome analysis, total DNA was extracted from cecal contents using the E.Z.N.A.® Soil DNA Kit (Omega Bio-Tek, USA) according to the manufacturer’s instruction. DNA concentration and purity was measured by NanoDrop2000. The V3-V4 variable region of the bacterial 16 S rRNA genes was amplified using the primers 338 F (5′- ACTCCTACGGGAGGCAGCAG-3′) and 806 R (5′- GGACTACHVGGGTWTCTAAT-3′). PCR products were mixed in equidensity ratios using GeneTools Analysis Software (version 4.03.05.0, SynGene) and subsequently purified with an AxyPrep DNA Gel Extraction Kit (Axygen, USA) in accordance with the manufacturer’s instructions. Sequencing libraries were prepared using the NEXTFLEX Rapid DNA-Seq Kit for Illumina (Bioo Scientific, USA) and then sequenced on a MiSeq PE300/NovaSeq PE250 platform (Shanghai Majorbio Technology Co., Ltd. Shanghai, China). Quality control and sequence splicing were performed using Fastp software (https://github.com/OpenGene/fastp, version 0.20.0) and FLASH software (http://www.cbcb.umd.edu/software/flash, version 1.2.7). UPARSE software (http://drive5.com/uparse/, version 7.1), clustered sequence into OTUs at 97%. In order to minimize the impact of sequencing depth on the subsequent alpha and beta diversity data analysis, all samples was normalized to 20,000 sequences. After flattening, the average sequence coverage amount to 99.09%. Taxonomic annotation of OTU species was performed using the RDP classifier (http://rdp.cme.msu.edu/, version 2.11) and compared with the Silva 16S rRNA gene database (v138) with a 70% confidence threshold. The community composition of each sample was calculated at the different species classification levels. Bioinformatics analysis of the gut microbiota was performed using the Majorbio Cloud platform (https://cloud.majorbio.com).

### Isolation and identification of fecal bacteria strains

Fresh feces collected from mice fed a high-iron diet were dissolved with 0.1% L-cysteine, and the homogenates were serially diluted and coated on blood plates. After anaerobic culture at 37 °C for 72 h, monoclonal colonies with different morphologies were selected and dissolved in the BHI medium supplemented with 0.1% cysteine, vitamin K (Hopebio, China), heme chloride (Hopebio), 10% FBS, and incubated anaerobically at 37 °C for 18 h. Bacterial DNA was extracted using a TIANamp bacterial DNA kit (TIANGEN). To identify the isolated bacterial strains, 16S rRNA gene sequences were amplified by PCR using the 16S rRNA gene primer pairs 27F (5’-AGAGTTTGATCCTGGCTCAG-3’) and 1492 R (5’-GGTTACCTTGTTACGACTT-3’). The resulting sequences were compared to the sequences in NCBI BLAST. The full-length 16S rRNA sequence of the identified strains was compared with all OTU sequences in the second-generation sequencing to screen out the dominant strains in mice fed a high-iron diet.

### Metabolites extraction and LC-MS/MS analysis

To extract metabolites, 50 mg of cecal content samples were accurately weighed, and treated with a 400 µL of methanol: water (4:1, v/v) solution. The mixture was allowed to settled at − 20°C and treated with a high-throughput tissue crusher Wonbio-96c at 50 Hz for 6 min, then vortex for 30 s and subjected to ultrasound at 40 kHz for 30 min at 5 °C. After that, the samples were placed at − 20 °C to precipitate proteins for 30 min. The supernatants were carefully transferred to sample vials for LC-MS analysis after centrifugation at 13,000 × g for 15 min at 4 °C.

Chromatographic separation of metabolites was performed using a Thermo UHPLC system equipped with an ACQUITY BEH C18 column (100 mm × 2.1 mm i.d., 1.7 µm; Waters, Milford, USA). Then mass spectrometric data were collected by a Thermo UHPLC-Q Exactive HF-X Mass Spectrometer equipped with an electrospray ionization source operating in both positive and negative ion modes. After UPLC-TOF/MS analyses, the raw data were imported into Progenesis QI 2.3 (Nonlinear Dynamics, Waters, USA) for peak detection and alignment.

The data were analyzed using the Majorbio cloud platform (www.cloud.majorbio.com). The processing results generated a data matrix including retention time, mass-to-charge ratio values, and peak intensity. MS/MS fragment spectra and isotope ratio differences were obtained by searching reliable biochemical databases, such as the Human Metabolome Database (http://www.hmdb.ca/) and the Metlin database (https://metlin.scripps.edu/). Multivariate statistical analysis was performed using the ropls (version 1.6.2, http://bioconductor.org/packages/release/bioc/html/ropls.html) R package from Bioconductor on the Majorbio Cloud Platform.

### *In vitro* co-culture

For the *in vitro* assay with *C. difficile* and ferrous sulfate, *C. difficile* was cultured two days on BHI agar in anaerobic chamber at 37 °C. After that, the bacterial concentration was adjusted to 1 × 10^5^ CFU/mL in a final volume of 5 mL of BHI broth, with or without ferrous sulfate at 10 or 5 mg/mL. Cultures were incubated for 24 h anaerobically at 37 °C. Similarly, proline or TUDCA was co-cultured with 1 × 10^5^ CFU/mL *C. difficile* at 0.1 of 1 mg/mL. Cultures were incubated for 18 h. *C. difficile* CFU were detected 24 h after plating on BHI agar, and TcdB was detected according to the manufacturer’s instructions.

For the *in vitro* co-culture of *C. difficile* and the three bacterial strains, the concentration of *C. difficile* was adjusted and maintained at 1 × 10^5^ CFU/mL in a final volume of 2 mL of BHI broth, with or without *E. coli AVS0501*, *E. fergusonii PW6* or *E. coli IRQBAS57* at 1 × 10^5^ or 1 × 10^6^ CFU/mL. *C. difficile* CFU were detected at 24 h after plating on BHI agar.

### Statistical analysis

Statistical analyses were performed using GraphPad Prism version 9.5 (GraphPad Software, San Diego, CA, USA). Scores, weights, and assay values were analyzed using a one-way or two-way Repeated Measures Analysis of Variance (ANOVA) test, multiple t-tests, or Student’s two-tailed t-test where appropriate. Data are presented as mean ± standard error of the mean (SEM). Values of *p < 0.05, **p < 0.01, ****p < 0.0001 were considered statistical significance.

## Results

### High dietary iron protects mice against CDI

**Figure 1.**
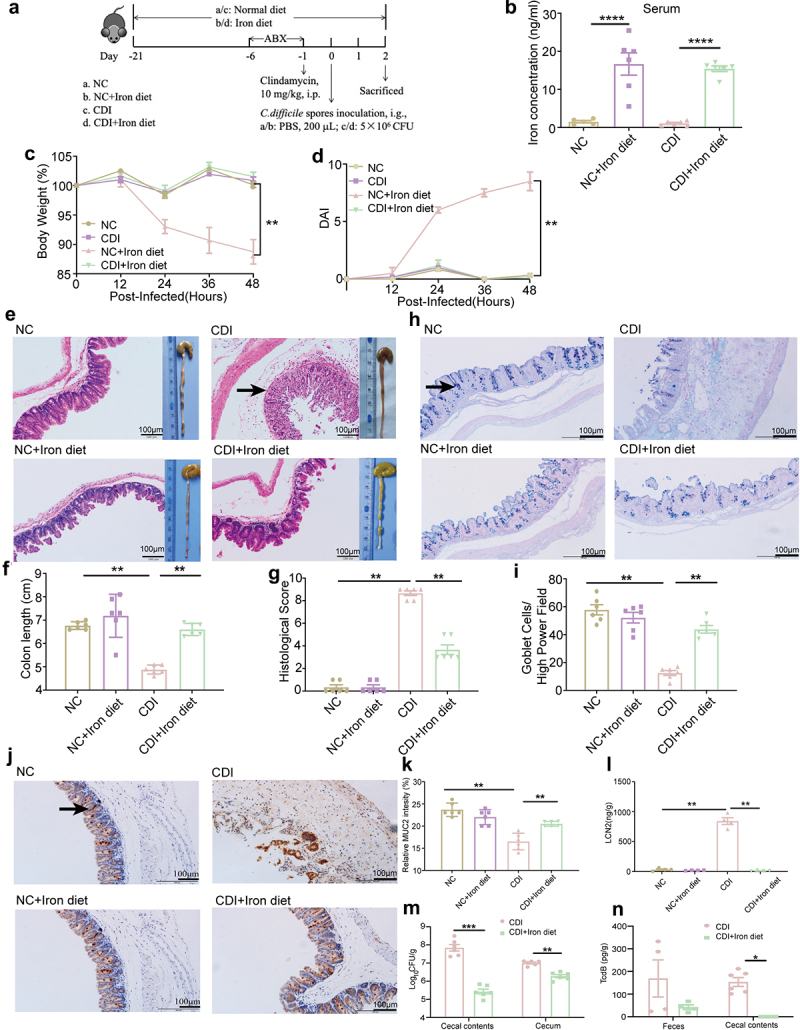
Efficacy of the high dietary iron against CDI. (a) C57BL/6J mice were fed with the normal or high iron (400 mg/kg ferrous sulfate) diet before and throughout the experiment for three weeks (n = 6). Mice were treated with the antibiotic mixture (ABX) for 5 d and then received a single i.P. dose of clindamycin (10 mg/kg). 1 day later, mice were infected with 5 × 10^6^ CFU of *C. difficile* spores (day 0). Serum, cecum, colon, cecal contents, and feces were taken from the sacrificed mice at 48 hpi. (b) Determination of the serum iron concentration using the iron assay kit. (c-d) Mice were monitored for body weight change (c) and DAI (d) at 12 hpi, 24 hpi, 36 hpi and 48 hpi. (e) Macroscopic photos of colon and representative HE-staining images (200×) of cecum. Scale bar: 100 μm. Arrow indicates the infiltration of inflammatory cells. (F) measurement and quantification of colon length. (g) Histological score of cecal tissues collected from the indicated mice. (h) Representative goblet cell staining images (200×) of cecum. Scale bar: 100 μm. Arrow indicates goblet cells. (i) Quantification of cecal goblet cells. (j) representative MUC2 immunohistochemical staining images (200×) of colon. Scale bar: 100 μm. Arrow indicates MUC2. (k) relative expression intensity of MUC2. (l) LCN2 levels were determined in the cecal contents using ELISA (n = 4). (m) the bacterial loads in cecum and cecal contents at 48 hpi (n = 6). (n) the level of *C. difficile* TcdB in cecal contents and feces at 48 hpi (n = 4–6). Data are shown as the mean ± SEM. Statistical significance was determined by two-way ANOVA (C and D) or one-way ANOVA (b,f,g,i,k,l) or multiple t tests (m,n), *p< 0.05, **p< 0.01, ***p< 0.001.

During intestinal inflammation, host nutritional immunity starves microbes from essential micronutrients such as iron. Studies have reported that iron levels are reduced in patients infected with *C. difficile* [23]. To investigate whether high dietary iron can improve host resistance to CDI, mice were fed dietary iron (400 mg/kg ferrous sulfate) or a normal diet (100 mg/kg ferrous sulfate) for three weeks before infection (Figure 1a). Upon infection, mice fed a high iron diet exhibited higher serum iron accumulation than mice fed a normal diet at 48 hpi (Figure 1b). Dietary iron treatment significantly alleviated body weight loss and decreased disease activity index (DAI) scores induced by CDI (Figure 1c,d). Compared to the NC group, cecum tissues in the CDI group showed severe intestinal epithelial injury, recess atrophy or loss, mucosal edema, and substantial inflammatory cell infiltration (Figure 1e), with significantly shortened colon length (Figure 1f) and the highest histological scores (Figure 1g), whereas the CDI+Iron diet group exhibited relatively less intestinal epithelial damage, diminished inflammatory cell infiltration and mucosal swelling, and significantly reduced colon shortening and lower histological scores (Figure 1e–g). Furthermore, the iron-treated CDI mice showed a comparatively intact intestinal epithelium with more goblet cells than CDI mice (Figure 1h,i). MUC2, the mucin secreted by goblet cells, was significantly higher in the CDI+Iron diet group than in the CDI group (Figure 1j,k). In addition, the amount of fecal LCN2, a noninvasive inflammatory marker, was significantly lower upon high-iron treatment (Figure 1l). These results demonstrated that dietary iron alleviates CDI-induced inflammation and intestinal damage. In addition, the amount of *C. difficile* in the cecal contents and cecum and the TcdB titers in cecal contents and feces were both decreased upon iron treatment (Figure 1m,n), indicating that dietary iron decreases the pathogenesis of *C. difficile* by reducing bacterial colonization and toxin production.

Additionally, mice were given 0.4 mg/mL vancomycin for 5 days after the primary infection to induce recurrent CDI (Figure S1a). Iron-treated rCDI mice showed less body weight loss and lower DAI scores than rCDI mice (Figure S1b-c). In addition, cecum tissue showed less intestinal epithelial damage, diminished inflammatory cell infiltration, and mucosal edema (Figure S1D), accompanied by slightly less colon shortening and a significantly lower histological score upon iron treatment (Figure S1e-f). Taken together, these data indicate that high dietary iron was effective at protecting against primary CDI, as well as preventing CDI relapse after vancomycin treatment.

### High dietary iron alleviates intestinal immune response in CDI mice

**Figure 2.**
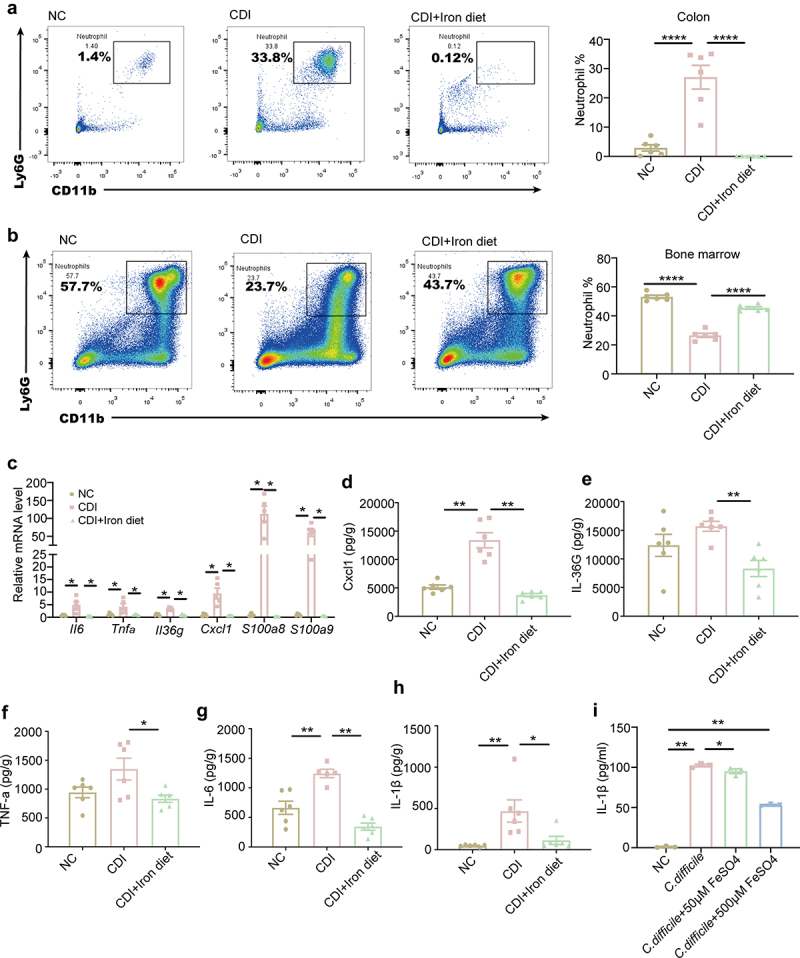
High dietary iron reduced the inflammatory response in CDI. C57BL/6J mice were fed with the normal or high iron diet before and throughout the experiment. On day 0, each mouse in CDI group and CDI+Iron diet group was given *C. difficile* spores. Cecum, colon and femurs were taken from the sacrificed mice at 48 hpi for further analysis. (a-b) representative flow cytometry graphs and the percentages of neutrophils in the colonic lamina propria (a) and bone marrow (b) (n = 6). (c) the mRNA expression levels of *Il6*, *Tnfa*, *Il36g*, *Cxcl1*, *S100a8* and *S100a9* in the colon were determined using qPCR (n = 5). (d-h) the concentration of CXCL1 (d), IL-36 G (e), TNF-α (f), IL-6 (g), and IL-1β (h) in the cecum were determined using ELISA (n = 6). (I) *in vitro* IL-1β production by neutrophils after priming with ferrous sulfate followed by stimulation with *C. difficile* (n = 3). Data are the mean ± SEM. Statistical significance was determined by one-way ANOVA (a-b and d-i) or multiple t tests (c), *p< 0.05, **p< 0.01, ***p< 0.0001.

Immune response is a key driver of CDI onset and recovery [29]. To evaluate the effect of dietary iron on CDI, the immune cell population in the colonic lamina propria and levels of cytokines in the cecum and colon were analyzed. CDI significantly promoted neutrophil recruitment into the colonic lamina propria, and a high-iron diet reduced the percentage of neutrophils that accumulated in the colon of CDI mice (Figure 2a). The increased colonic neutrophils in CDI may mobilize from the bone marrow, as the proportion of neutrophils in the bone marrow of CDI mice decreased significantly, whereas dietary iron treatment restored this reduction (Figure 2b). Furthermore, the expression of *Il6*, *Tnfa*, *Il36g*, *Cxcl1*, *S100a8*, and *S100a9* significantly increased in CDI mice, while dietary iron reduced this increase (Figure 2c). In accordance with this, the levels of pro-inflammatory factors, such as CXCL1, IL-36 G (IL-36γ, TNF-α, IL-6, and IL-1β, were significantly increased in the CDI group and decreased in the CDI +Iron diet group (Figure 2d–h), suggesting that high iron effectively prevented the recruitment of neutrophils to the infection site, thus reducing the inflammatory response. To investigate whether high iron levels could directly affect neutrophil function, bone marrow-derived neutrophils were obtained and seeded into 6-well plates (1 × 10^5^ cells/mL). Initially, cells were incubated with 50 μM and 500 μM ferrous sulfate for 2 h. *C. difficile* (1 × 10^7^ CFU/mL) was then added to the culture for the next 4 h. Subsequently, supernatants were collected and IL-1β was quantified by ELISA. As shown in Figure 2i, *C. difficile* induced a large amount of IL-1β secretion by neutrophils, while both 50 μM and 500 βM FeSO4 inhibited *C. difficile*-induced IL-1β secretion. Collectively, our results suggest that dietary iron may alleviate CDI by reducing neutrophil recruitment and secretion of inflammatory cytokines

### High dietary iron alters gut microbiota to alleviate CDI

To study the effect of high iron on the gut microbiota, mice were treated with high dietary iron (400 mg/kg ferrous sulfate) for three weeks, after which the cecal contents were collected and analyzed by 16S rRNA gene sequencing. Principal component analysis (PCA) and principal coordinate analysis (PCoA), which were used to assess beta diversity, revealed that bacteria in mice treated with high iron were different from those in normal mice (Figure S2A-B). According to the alpha diversity indices, compared with the NC group, the species richness (Ace index) of the iron diet group was significantly increased, while the species diversity (Shannon index) was slightly increased (Figure S2C-D). The dominant phyla in the NC group were *Bacteroidota*, whereas the iron diet group was rich in *Firmicutes* (Figure S2E). The dominant families in the NC group were *Muribaculaceae* and *Lactobacillaceae*, whereas *Oscillospiraceae* and *Akkermansiaceae* were dominant in the iron diet group (Figure S2F). At the genus level, 11 genera (*Lachnospiraceae*, *Oscillospiraceae*, *Desulfovibrionaceae*, *Akkermansia*, *Parabacteroides*, *Colidextribacter*, *Mucispirillum*, and Mucispirillum) were higher in the iron diet group than in the NC group, and five genera (*Muribaculaceae*, *Lactobacillus*, *Desulfovibrio* and *Alistipes*) were decreased (Figure S2G). The dominant species in the NC group were mainly from *Muribaculaceae*, whereas the dominant species in the iron diet group were mainly from *Lachnospiraceae*, *Oscillospiraceae* and *Akkermansia* (Figure S2H). *Muribaculaceae* and *Akkermansiaceae* have previously been identified as potential mucus degraders [30] and are closely related to *C. difficile* colonization resistance.

**Figure 3.**
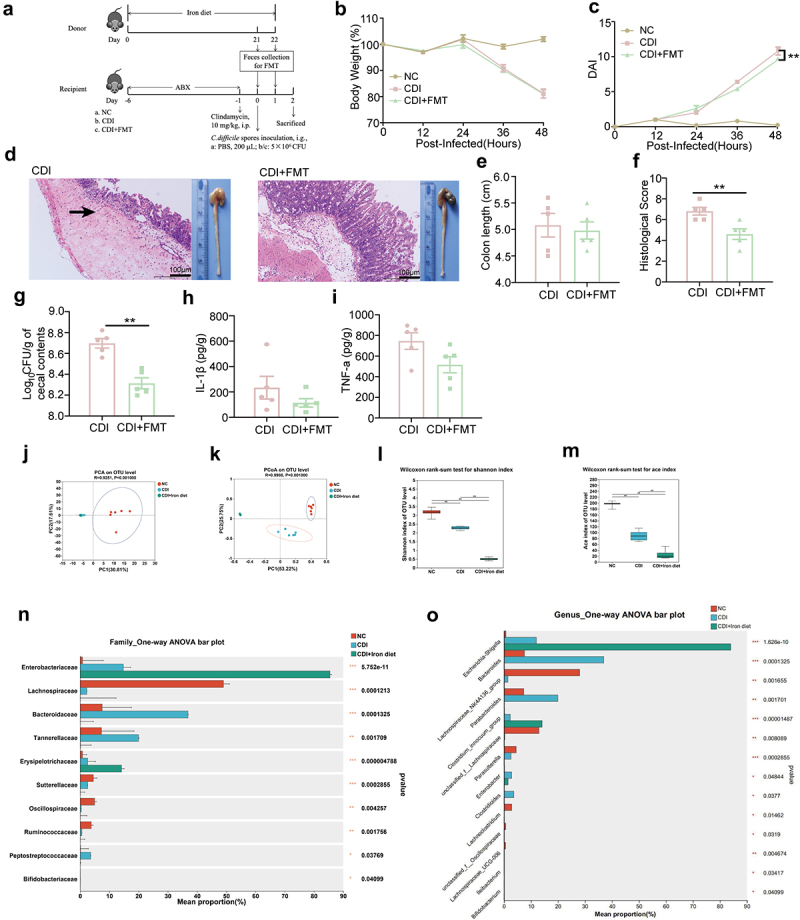
High iron diet significantly altered the gut microbiota in CDI mice. (a) schematic image illustrating FMT design. The donor mice were fed with the high iron diet (400 mg/kg ferrous sulfate) for 3 weeks. The recipient mice were treated with ABX for 5 days and then received a single i.P. dose of clindamycin (10 mg/kg). one day later, mice were infected with 5 × 10^6^ CFU of *C. difficile* spores (day 0). At the same time, feces from donor mice were collected and given to recipient mice by oral gavage for two days. Cecum and colon were taken from the sacrificed mice at 48 hpi (n = 5). (b and c) mice were monitored for body weight change (b) and DAI (C) at 12 hpi, 24 hpi, 36 hpi and 48 hpi. (d) macroscopic images of colon and representative HE-staining images (200×) of cecum. Scale bar: 100 μm. Arrow indicates the infiltration of inflammatory cells. (e) measurement and quantification of colon length. (f) histological score of cecal tissues collected from the indicated mice. (g) the bacterial loads in the cecal contents at 48 hpi (n = 5). (h-i) the concentrations of IL-1β (h) and TNF-α (I) in the cecum were determined using ELISA (n = 5). (j-o) C57BL/6J mice were fed with the normal or high iron diet before and throughout the experiment. On day 0, each mouse in CDI group and CDI+Iron diet group was given *C. difficile* spores. Cecal contents from NC, CDI and CDI+Iron diet groups were collected and analyzed by 16S rRNA gene sequencing (n = 6). (j) unweighted principal component analysis (PCA) score plots of OTUs. (K) unweighted principal coordinate analysis (PCoA) score plots of OTUs. (l) Shannon index. (M) Ace index. (N) multi species comparison bar chart of family. (o) multi species comparison bar chart of genus. Data are the mean ± SEM. Statistical significance was determined by two-way ANOVA (b and c), Mann-Whitney test (E-I), Wilcoxon rank-sum test (L and M) or one-way ANOVA (N and O). *p < 0.05; **p < 0.01; ***p < 0.001.

FMT can effectively treat CDI [31], so we investigated whether the gut microbiota from iron diet mice could alleviate CDI. First, the donor mice were fed with high dietary iron for three weeks, and then the recipient mice received FMT from donor mice on the day of infection and 24 hpi (Figure 3a). Mice in the CDI+FMT group displayed comparable body weight (Figure 3b), but a lower DAI than the CDI mice (Figure 3c). In addition, mice that received FMT had more intact intestinal epithelium, less inflammatory cell infiltration, and mucosal edema (Figure 3d), accompanied by comparable colon length but significantly lower histological scores than CDI mice (Figure 3e,f). The amount of *C. difficile* in the cecal contents apparently decreased upon FMT (Figure 3g), and the secretion of IL-1β and TNF-α was slightly lower after FMT (Figure 3h-i). Taken together, these results indicate that a high-iron diet may alleviate CDI by altering gut microbiota.

### E. coli AVS0501 protects mice against CDI

To further explore the effect of the high-iron diet on the gut microbiota in CDI mice, the cecal contents were collected at 48 hpi and analyzed by 16S rRNA gene sequencing. PCA and PCoA showed that the gut microbiota of the CDI+Iron diet group was different from that of the NC and CDI groups (Figure 3j-k). Shannon and ace indices showed that species diversity and abundance were highest in the NC group, followed by the CDI and CDI+Iron diet groups (Figure 3l-m). The dominant families were *Lachnospiraceae* in the NC group, *Bacteroidaceae* in the CDI group, and *Enterobacteriaceae* in the CDI + Iron diet group (Figure 3n). At the genus level, *Escherichia-Shigella* was significantly increased in the CDI+Iron diet group, whereas *Lachnospiraceae* and *Bacteroides* were the dominant bacterial genera in the NC and CDI groups, respectively (Figure 3o). Therefore, we wondered whether *Escherichia-Shigella* genus plays a role in high dietary iron alleviated CDI.

**Figure 4.**
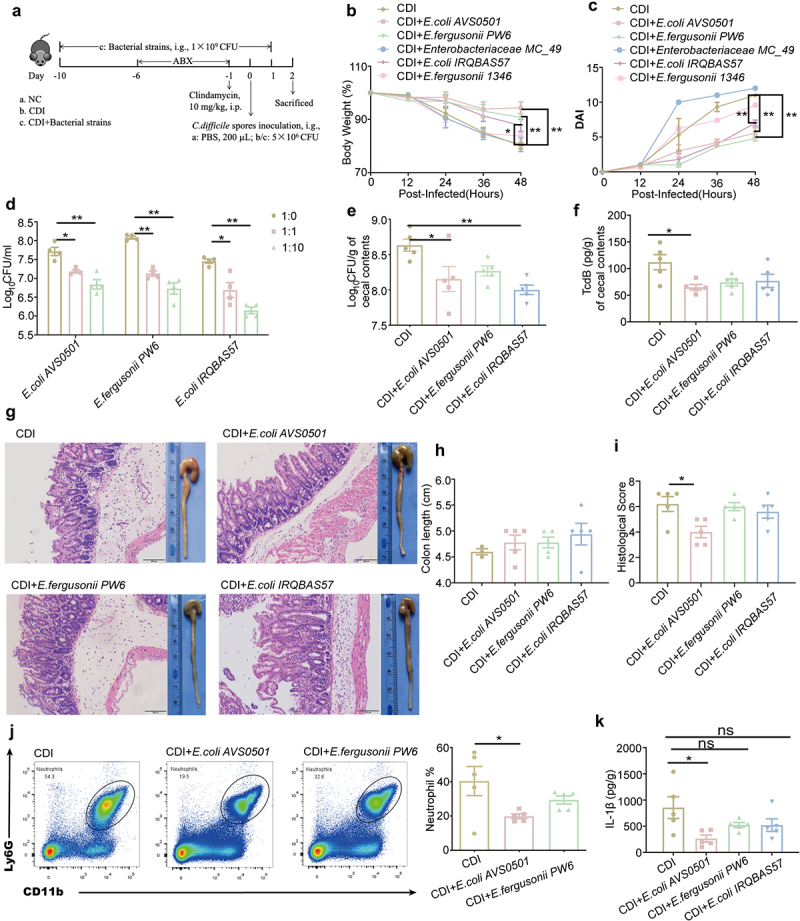
The isolated bacterial strains from mice fed on high iron diet protected mice from CDI. (a) strategy diagram of bacterial strains isolated from mice fed on the high iron diet for treating CDI. C57BL/6J mice were given PBS or bacterial strains for 12 days before and throughout the experiment. Mice were treated with the ABX for 5 d and then received a single i.P. dose of clindamycin (10 mg/kg). 1 d later, mice were infected with 5 × 10^6^ CFU of *C. difficile* spores (day 0). Cecum and colon were taken from the sacrificed mice at 48 hpi (n = 5). (b-c) Mice were monitored for weight change (b), and DAI (c) at 12 hpi, 24 hpi, 36 hpi and 48 hpi. (d) the *C. difficile* were incubated with the three indicated bacterial strains at different ratios (n = 4) *in vitro* for 24 h, and the *C. difficile* loads were evaluated. (e) the bacterial loads in cecal contents at 48 hpi (n = 5). (f) the level of *C. difficile* TcdB in cecal contents at 48 hpi (n = 5). (g) Macroscopic findings of colon and representative HE-staining images (200×) of cecum. Scale bar: 100 μm. Arrow indicates the infiltration of inflammatory cells. (h) measurement and quantification of colon length. (i) histological score of cecal tissues collected from the indicated mice. (j) representative flow cytometry graphs and the percentages of neutrophils in the colonic lamina propria at 48 hpi (n = 5). (k) the concentration of IL-1β in the cecum was determined using ELISA. Data are the mean ± SEM. Statistical significance was determined by two-way ANOVA (B and C) or multiple t tests (D) or one-way ANOVA (e,f,h,i,j,k), ns, no significance. *p< 0.05, **p< 0.01.

Fresh feces collected from mice fed a high-iron diet were dissolved, homogenized, serially diluted, and coated on blood plates. After anaerobic culture at 37°C for 72 h, monoclonal colonies with different morphologies were selected, cultured anaerobically at 37°C for 18 h and identified by using 16S rRNA sequencing. Among bacterial strains isolated and identified, a variety of strains belong to the *Escherichia-Shigella* genus were identified, such as *Escherichia coli* strain *AVS0501* (*E. coli AVS0501*), *Escherichia fergusonii* strain *PW6* (*E. fergusonii PW6*), *Enterobacteriaceae* bacterium *MC_49 Shigella* (*Enterobacteriaceae MC_49*), *Escherichia coli IRQBAS57* (*E. coli IRQBAS57*), *Escherichia fergusonii* strain *1346* (*E. fergusonii 1346*) and so on. Next, these five strains were selected to treat the CDI mice (Figure 4a). Surprisingly, three strains, *E. coli AVS0501*, *E. fergusonii PW6*, and *E. coli IRQBAS57* significantly alleviated body weight loss and decreased the DAI in mice with CDI (Figure 4b-c). Next, the direct antibacterial effects of these three strains were explored, coculture of *C. difficile* with the three strains separately showed that both of *E. coli AVS0501*, *E. fergusonii PW6*, and *E. coli IRQBAS57* inhibited the growth of *C. difficile in vitro* (Figure 4d). Furthermore, the *C. difficile* loads in cecal contents were examined. *E. coli AVS0501* and *E. coli IRQBAS57* significantly decreased the colonization of *C. difficile* in *vivo* (Figure 4e), and *E. coli AVS0501* significantly reduced the production of toxin TcdB (Figure 4f). Compared to the CDI group, *E. coli AVS0501* treatment reduced cecal epithelial injury, mucosal edema, and inflammatory cell infiltration caused by CDI, whereas the other two strains displayed comparable results (Figure 4g). Although the colon length in each group was similar, the degree of pathological damage was significantly reduced in the CDI+*E. coli AVS0501* group (Figure 4h-i). Moreover, *E. coli AVS0501* significantly inhibited neutrophil recruitment into the colonic lamina propria (Figure 4j) and reduced the secretion of IL-1β (Figure 4k). Taken together, these results suggest that the identified *E. coli AVS0501* strain play an important role in protecting mice against CDI.

### Effects of high dietary iron on intestinal metabolism in CDI mice

**Figure 5.**
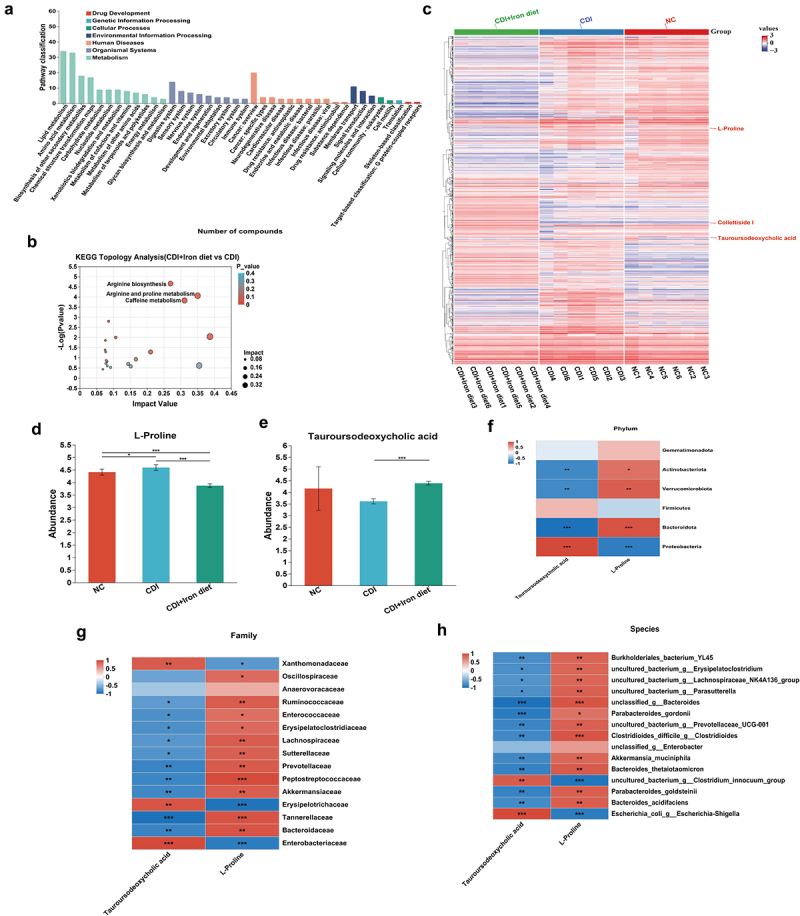
Effects of high iron diet on intestinal metabolism. C57BL/6J mice were fed with the normal or high iron diet before and throughout the experiment. On day 0, each mouse in CDI group and CDI+Iron diet group was given *C. difficile* spores. Cecal contents from NC, CDI and CDI+Iron diet groups were collected for untargeted metabolomics analyses (n = 6). (a) analysis of KEGG functional pathway. (b) the topological analysis of KEGG pathway of differential metabolites between NC, CDI and CDI +iron diet group. (c) the heatmap of differential metabolites between NC, CDI and CDI +iron diet group. (d-e) the abundance of L-Proline (d) and TUDCA (E) in cecal contents. Data are the mean±SD. Statistical significance was determined by one way ANOVA (D and E). * p< 0.05, *** p< 0.001. (f-h) the correlation between intestinal metabolites and microbiota were analyzed based on Spearman correlation coefficient. (f) correlation matrix between the two metabolites and TOP 6 enriched bacteria phyla. (g) correlation matrix between the two metabolites and TOP 15 enriched bacteria families. (h) correlation matrix between the two metabolites and TOP 15 enriched bacteria species. The metabolites were annotated on the bottom, the bacteria were annotated at the right. Each grid represented the correlation between the two attributes, red indicates positive correlation, while blue indicates negative correlation, statistical significance was determined by Spearman correlation coefficient, *p < 0.05, **p < 0.01, ***p < 0.001.

Microbiota-derived metabolites can modulate host responses through functional interactions with other microbes in their ecological niche; thus, cecal contents were collected for untargeted metabolomic analysis. Metabolic pathway and topological analysis of pathways were performed according to KEGG metabolic pathways. Our results showed that the most enriched pathways in the CDI and CDI+Iron diet groups were lipid and amino acid metabolism (Figure 5a). Arginine biosynthesis and arginine and proline metabolism were the most significantly affected pathways in the CDI+Iron diet group compared to those in the CDI group (Figure 5b), with proline as the core metabolite. The abundance of L-proline was significantly increased in CDI mice compared to NC mice, but decreased in iron-treated CDI mice (Figure 5c-d). Furthermore, the abundance of TUDCA was higher in the CDI+Iron diet group than in the CDI group (Figure 5c,e). Spearman correlation analysis showed that changes in proline and TUDCA levels were consistent with changes in the gut microbiota of CDI+Iron-fed mice (Figure 5f-h). The most interesting finding was the significant correlations between these metabolites and *Escherichia coli* at the species level, that is, a positive correlation for TUDCA and a negative correlation for L-proline (Figure 5h). These results suggested that high dietary iron may influence the enrichment of L-proline and TUDCA to ameliorate CDI, which may be related to *E. coli AVS0501*.

### Decreased proline level reduces host susceptibility to CDI

**Figure 6.**
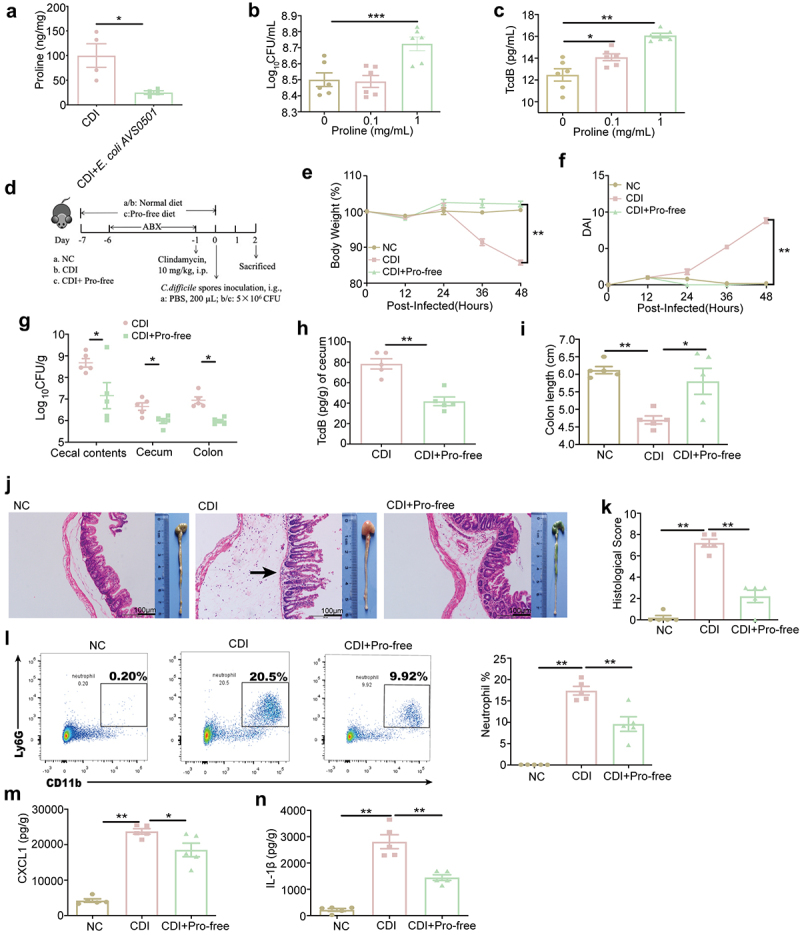
Proline-free diet alleviates *C. difficile* infective enteritis. (a) C57BL/6J mice were given PBS or *E. coli AVS0501* strains for 12 days before and throughout the experiment. Cecal contents were taken from the sacrificed mice at 48 hpi, and the level of L-proline in the cecal contents were analyzed. (b-c) *C. difficile* were incubated with 0.1 mg/mL and 1 mg/mL proline *in vitro* for 24 h (n = 6), the *C. difficile* load (B) and the toxin TcdB production (c) were evaluated. (d) Schematic image. C57BL/6J mice were fed with the normal or Pro-free diet for a week before *C. difficile* infection. Mice were treated with the ABX for 5 d and then received a single i.P. dose of clindamycin (10 mg/kg). 1 d later, mice were infected with 5 × 10^6^ CFU of *C. difficile* spores (day 0). Cecum, cecal contents and colon were taken from the sacrificed mice at 48 hpi (n = 5). (E-F) the body weight change (e), and DAI (f) were monitored at 12 hpi, 24 hpi, 36 hpi and 48 hpi. (g) the bacterial loads in cecal contents, cecum and colon at 48 hpi (n = 5). (h) the level of *C. difficile* TcdB in cecum at 48 hpi (n = 5). (i) measurement and quantification of colon length. (j) macroscopic findings of colon and representative HE-staining images (200×) of cecum. Scale bar: 100 μm. Arrow indicates the infiltration of inflammatory cells. (k) quantitation of histology score in cecum. (l) representative flow cytometry graphs and the percentages of neutrophils in the colonic lamina propria at 48 hpi (n = 5). (m-n) the levels of CXCL1 (M) and IL-1β (n) in the cecum were determined using ELISA (n = 5). Data are the mean ± SEM. Statistical significance was determined by Mann-Whitney test (a,h), one-way ANOVA (b,c,i k-n), two-way ANOVA (E and F) or multiple t tests (G), *p< 0.05, **p< 0.01, ***p< 0.001.

To evaluate whether the reduction of L-proline in the CDI+Iron diet group was related to *E. coli AVS0501*, the proline level in the cecal contents was detected, and the proline concentration in the CDI+*E. coli AVS0501* group was significantly lower than that in the CDI group (Figure 6a). It has been reported that CDI patients tend to decompose amino acids for energy, and proline plays an important role in *C. difficile* stickland metabolism [32,33]. *In vitro*, when the culture medium was supplemented with 1 mg/mL proline, the bacterial titers of *C. difficile* apparently raised (Figure 6b), and the supplementation of 0.1 mg/mL and 1 mg/mL proline significantly increased the TcdB production (Figure 6c). Furthermore, *in vivo*, mice were fed a proline-free (Pro-free) diet for a week before CDI (Figure 6d), and body weight and DAI were monitored. Compared to CDI mice, mice fed a Pro-free diet showed no clinical symptoms caused by CDI (Figure 6e-f); moreover, the *C. difficile* loads in the cecal contents, cecum, and colon were all decreased significantly (Figure 6g), along with apparently lower TcdB titers in the cecum (Figure 6h). Correspondingly, the Pro-free diet alleviated CDI-induced colon shortening, intestinal epithelial loss, and mucosal edema, accompanied by significantly lower histological scores (Figure 6i-k). Furthermore, the Pro-free diet decreased the recruitment of neutrophils into the colonic lamina propria (Figure 6l), and the intestinal levels of CXCL1 and IL-1β were apparently reduced in the CDI+Pro- free group compared to the CDI group (Figure 6m-n). Overall, these observations demonstrate that l-proline plays a critical role in host susceptibility to CDI.

### TUDCA treatment reduces host susceptibility to CDI

**Figure 7.**
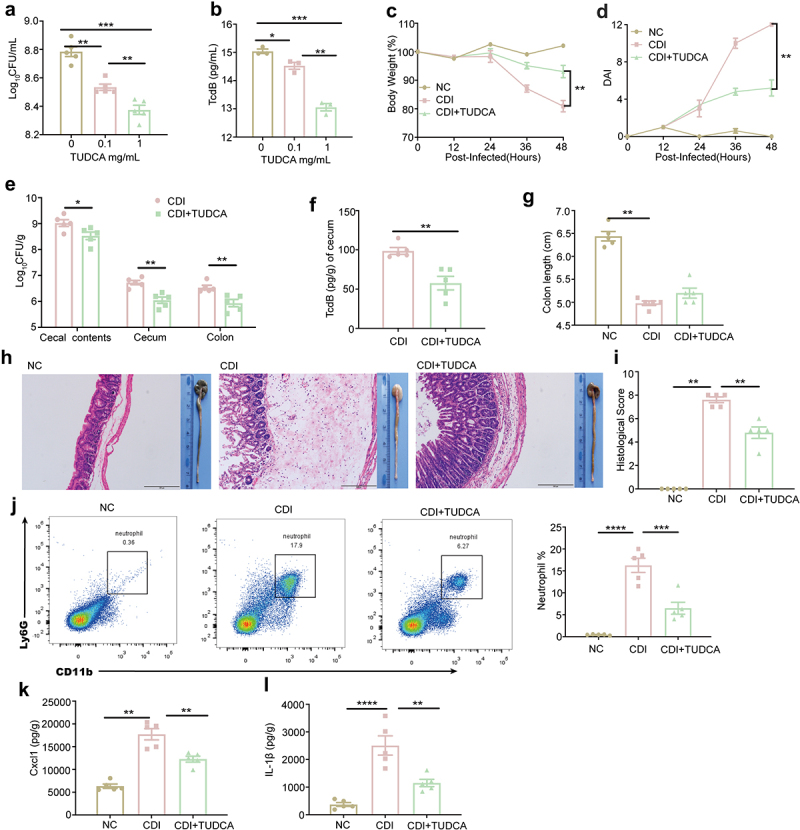
TUDCA treatment alleviates *C. difficile* infective enteritis. (a-b) *C. difficile* were incubated with 0.1 mg/mL and 1 mg/mL TUDCA *in vitro* for 18 h, the *C. difficile* load (A) and the toxin TcdB production (b) were evaluated (n = 3). (c-l) Mice in the NC and CDI groups received sterile water, while mice in the CDI+TUDCA group received 100 mg/kg TUDCA gavage one week before and throughout the experiment. Mice were treated with ABX for 5 days and then received a single i.P. dose of clindamycin. one day later, mice were infected with 5 × 10^6^ CFU of *C. difficile* spores (day 0). Cecum and colon were taken from the sacrificed mice at 48 hpi (n = 5). (c-d) mice were monitored for body weight change (c) and DAI (d) at 12 hpi, 24 hpi, 36 hpi and 48 hpi. (e) the bacterial loads in cecal contents, cecum and colon at 48 hpi (n = 5). (f) the level of *C. difficile* TcdB in cecum at 48 hpi (n = 5). (g) measurement and quantification of colon length. (h) macroscopic findings of colon and representative HE-staining images (200×) of cecum. Scale bar: 100 μm. Arrow indicates the infiltration of inflammatory cells. (i) quantitation of histology score in cecum. (j) representative flow cytometry graphs and the percentages of neutrophils in the colonic lamina propria at 48 hpi (n = 5). (k-l) the levels of CXCL1 (K) and IL-1β (l) in the cecum were determined by ELISA (n = 5). Data are the mean ± SEM. Statistical significance was determined by or one-way ANOVA (a-b, g, i-l), two-way ANOVA (c-d), multiple t tests (e), or Mann-Whitney test (f), *p< 0.05, **p< 0.01, ****p< 0.0001.

Similarly, the effect of TUDCA on CDI was explored. *In vitro*, the bacterial titers of *C. difficile* and the production of TcdB were progressively depressed when the culture medium was supplemented with 0.1 mg/mL and 1 mg/mL TUDCA (Figure 7a-b). Gavage of 100 mg/kg TUDCA was sufficient to protect mice against CDI, resulting in improved body weight loss and reduced DAI scores (Figure 7c-d). The *C. difficile* loads in the cecal contents, cecum, and colon were all decreased significantly (Figure 7e), along with lower TcdB production in the cecum (Figure 7f). Correspondingly, TUDCA treatment alleviated CDI-induced intestinal epithelial loss and mucosal edema, accompanied by significantly lower histological scores and comparable colon length when compared with the CDI group (Figure 7g-i). Furthermore, TUDCA treatment decreased the recruitment of neutrophils into the colonic lamina propria (Figure 7j), and cecal CXCL1 and IL-1β were apparently reduced in the CDI+TUDCA group compared to the CDI group (Figure 7k-l). These results indicate that a high-iron diet partially suppresses CDI by increasing intestinal TUDCA levels.

## Discussion

Overall, this study demonstrated the effectiveness of high dietary iron in alleviating CDI, which may have protective effects via three mechanisms: (1) inhibition of *C. difficile* colonization and TcdB production, (2) reducing the intestinal inflammatory response, and (3) regulating the gut microbiota, increasing the levels of TUDCA, and decreasing the levels of proline in the intestine. This sheds light on the interaction between the iron diet, gut microbiota and host immune system in reducing the severity of CDI.

Pathogens can acquire micronutrients by releasing metal-chelating molecules or utilizing high-affinity metal transporters to acquire divalent metal ions, such as iron, zinc, and manganese [34]. Iron is a nutrient resource for eukaryotic and prokaryotic cells. Excess iron and iron deficiency can affect health and infection outcomes. It has been reported that iron levels are reduced in CDI [23]. Interestingly, a high-iron diet (400 mg/kg iron) significantly reduced CDI compared to a normal-iron diet (100 mg/kg iron). The high-iron diet inhibited the colonization and toxin production of *C. difficile*; thus, intestinal mucosal damage was reduced, accompanied by increased intestinal goblet cells and MUC2 expression. Overall, mice that received sufficient iron beforehand were more resistant to *C. difficile.*

Disturbance of gut microbiota caused by antibiotics is the core of CDI. To clarify the underlying mechanism of the dietary iron-mediated reduction in the risk of CDI, the changes in gut microbiota composition following high-iron diet intervention were analyzed. Notably, the diversity of the gut microbiota in mice receiving high dietary iron was more abundant than that in normal mice. The dominant genera were *Muribaculaceae*, *Lactobacillus*, *Desulfovibrio* and *Alistipes* in the NC group, whereas *Lachnospiraceae*, *Oscillospiraceae*, *Desulfovibrionaceae*, *Akkermansia*, *Parabacteroides*, *Colidextribacter*, and *Mucispirillum* were dominant in the iron diet group. *Akkermansia muciniphila* can utilize mucin, which may be a key factor in its competitive inhibition of *C. difficile* colonization [33]. Moreover, *Oscillospiraceae* produces abundant short-chain fatty acids and secondary bile acids [35]. In this study, transplanting CDI mice with gut microbiota from mice fed a high-iron diet can reduce the severity of CDI, but because the addition of antibiotics in the infection model does not represent the pre-infection state of the microbiota. As FMT has been proven to be effective in treating CDI, this does not imply that dietary iron-altered gut microbiota plays a major role. Furthermore, the composition of the gut microbiota during CDI was analyzed. Interestingly, compared with normal mice, the diversity of gut microbiota in high-iron-fed mice were significantly reduced – specifically, their microbial species evenness and richness were even lower than those in CDI mice. The gut microbiota in the CDI+Iron diet group was predominantly composed of *Enterobacteriaceae, Escherichia-Shigella* genus. Several bacterial strains of *Enterobacteriaceae* have been isolated from the feces of CDI mice treated with dietary iron. After treating CDI mice with these bacterial strains, *E. coli AVS0501*, *E. fergusonii PW6* and *E. coli IRQBAS57* alleviated the clinical symptoms of CDI, among which *E. coli AVS0501* significantly reduced *C. difficile* colonization and toxin production, thus maintaining intestinal homeostasis. These findings have important implications for the treatment and prevention of CDI and may contain protective species.

Microbial-derived metabolites exhibit potent and specific biological activities and mediate systemic and local biological effects [36]. FMT regulates intestinal flora metabolites, including bile acids and short-chain fatty acids, to treat CDI [37]. Thus, untargeted metabolomics analyses were performed, and most amino acid metabolism pathways were significantly reduced in high iron-treated mice and correlated with the changed microbiota. Typically, colonization resistance developed by gut microbiota is not conducive to *C. difficile* colonization and proliferation [38,39]. Patients with CDI exhibit a chemical signature of Stickland amino acid fermentation, suggesting that *C. difficile* preferentially catabolizes branched-chain amino acids during infection [40]. Proline is an important substrate for *C. difficile* Stickland fermentation [41]. Proline levels were consistently decreased in the CDI+Iron diet group compared with the CDI group, and *E. coli AVS0501* colonization also reduced proline levels in CDI mice. Thus, it is possible that dietary iron reduces proline levels by increasing the abundance of *E. coli AVS0501* in CDI mice, warranting further analysis of their role in *C. difficile* pathogenesis. Furthermore, our data showed that *C. difficile* colonization was significantly reduced when mice were fed a Pro-free diet, thus alleviating the intestinal inflammatory response, including neutrophil infiltration and secretion of pro-inflammatory factors. Moreover, many metabolites, such as TUDCA, were also increased in high iron-treated CDI mice. TUDCA was reported to inhibit the effect of *C. difficile* toxin on Caco-2 cells *in vitro* [42,43]. TUDCA effectively relieved the clinical symptoms of CDI and reduced *C. difficile* colonization and toxin production. Spearman correlation analysis showed that *E. coli* was closely related to L-proline and TUDCA, and our results demonstrated that *E. coli AVS0501* colonization affected proline levels in CDI mice. Clearly, there is still much to be learned about the complex interplay between gut microbiota, metabolites, and *C. difficile.*

Collectively, our study provides evidence that high-iron diet intervention reduces susceptibility to CDI by regulating gut microbiota and metabolites. Moreover, due to the harsh nutritional requirements of *C. difficile*, the intestinal metabolic environment created by high-iron treatment can inhibit the colonization and growth of *C. difficile*. Therefore, the association between nutrient metabolism and CDI needs to be further explored in the future.

## Supplementary Material

Figure S1.jpg

Supplementary information.docx

Figure S2.jpg

ARRIVE Author Checklist.pdf

## Data Availability

The Mass spec-based metabolomics data generated in this study have been deposited in the MetaboLights database (MTBLS11336, https://www.ebi.ac.uk/metabolights/MTBLS11336). The 16S rRNA-seq data have been deposited in the NCBI Sequence Read Archive database (SRA, PRJNA1170229, https://www.ncbi.nlm.nih.gov/bioproject/PRJNA1170229). The datasets generated during and/or analyzed during the current study are available in the Figshare repository. https://dx.doi.org/10.6084/m9.figshare.29256140.

## References

[1] Martin JS, Monaghan TM, Wilcox MH. Clostridium difficile infection: epidemiology, diagnosis and understanding transmission. Nat Rev Gastroenterol Hepatol. 2016;13(4):206–19. doi: 10.1038/nrgastro.2016.25 26956066

[2] Lee CC, Lee JC, Chiu CW, et al. Clinical significance of toxigenic clostridioides difficile growth in stool cultures during the era of nonculture methods for the diagnosis of C. difficile infection. Microbiol Spectr. 2021;9(2):e0079921. doi: 10.1128/Spectrum.00799-21 34668727 PMC8528117

[3] Peery AF, Crockett SD, Murphy CC, et al. Burden and cost of gastrointestinal, liver, and pancreatic diseases in the United States: update 2018. Gastroenterology. 2019;156(1):254–272.e211. doi: 10.1053/j.gastro.2018.08.063 30315778 PMC6689327

[4] Kwak S, Choi J, Hink T, et al. Impact of investigational microbiota therapeutic RBX2660 on the gut microbiome and resistome revealed by a placebo-controlled clinical trial. Microbiome. 2020;8(1):125. doi: 10.1186/s40168-020-00907-9 32862830 PMC7457799

[5] Cold F, Svensson CK, Petersen AM, et al. Long-term safety following faecal microbiota transplantation as a treatment for recurrent clostridioides difficile infection compared with patients treated with a fixed bacterial mixture: results from a retrospective cohort study. Cells. 2022;11(3):435. doi: 10.3390/cells11030435 35159245 PMC8834574

[6] Fachi JL, de Oliveira S, Trsan T, et al. Fiber- and acetate-mediated modulation of MHC-II expression on intestinal epithelium protects from clostridioides difficile infection. Cell Host Microbe. 2025;33(2):235–251.e237. doi: 10.1016/j.chom.2024.12.017 39826540 PMC11974464

[7] Abt MC, Pt M, Pamer EG. Clostridium difficile colitis: pathogenesis and host defence. Nat Rev Microbiol. 2016;14(10):609–620. doi: 10.1038/nrmicro.2016.108 27573580 PMC5109054

[8] Sehgal K, Cifu AS, Khanna S. Treatment of clostridioides difficile infection. JAMA. 2022;328(9):881. doi: 10.1001/jama.2022.12251 35939317

[9] Ott SJ, Waetzig GH, Rehman A, et al. Efficacy of sterile fecal filtrate transfer for treating patients with clostridium difficile infection. Gastroenterology. 2017;152(4):799–811.e797. doi: 10.1053/j.gastro.2016.11.010 27866880

[10] McMillan AS, Zhang G, Dougherty MK, et al. Metagenomic, metabolomic, and lipidomic shifts associated with fecal microbiota transplantation for recurrent clostridioides difficile infection. mSphere. 2024;9(10):e0070624. doi: 10.1128/msphere.00706-24 39377587 PMC11520286

[11] Mori N, Hirai J, Ohashi W, et al. Clinical efficacy of Fidaxomicin and oral metronidazole for treating clostridioides difficile infection and the associated recurrence rate: a retrospective cohort study. Antibiotics (Basel). 2023;12(8):1323. doi: 10.3390/antibiotics12081323 37627743 PMC10451525

[12] Yang H, Wang L, Villafuerte Gálvez JA, et al. Clostridium difficile and gut health: bacteria, the gut microbiome, and diet. iMetaomics. 2024;2(1). doi: 10.1002/imo2.50 PMC12806537 41675702

[13] Mefferd CC, Bhute SS, Phan JR, et al. A high-Fat/High-protein, Atkins-type diet exacerbates clostridioides (clostridium) difficile infection in mice, whereas a high-carbohydrate diet protects. mSystems. 2020;5(1). doi: 10.1128/msystems.00765-19 PMC7018531 32047064

[14] Yakabe K, Higashi S, Akiyama M, et al. Dietary-protein sources modulate host susceptibility to clostridioides difficile infection through the gut microbiota. Cell Rep. 2022;40(11):111332. doi: 10.1016/j.celrep.2022.111332 36103838

[15] Zackular JP, Moore JL, Jordan AT, et al. Dietary zinc alters the microbiota and decreases resistance to clostridium difficile infection. Nat Med. 2016;22(11):1330–1334. doi: 10.1038/nm.4174 27668938 PMC5101143

[16] Loureiro AV, Barbosa MLL, Morais M, et al. Host and clostridioides difficile-response modulated by micronutrients and glutamine: an overview. Front Nutr. 2022;9:849301. doi: 10.3389/fnut.2022.849301 35795588 PMC9251358

[17] Raut AK, Hiwale KM. Iron deficiency anemia in pregnancy. Cureus. 2022;14:e28918. doi: 10.7759/cureus.28918 36225459 PMC9541841

[18] Ellwanger JH, Ziliotto M, Kulmann-Leal B, et al. Iron deficiency and soil-transmitted helminth infection: classic and neglected connections. Parasitol Res. 2022;121(12):3381–3392. doi: 10.1007/s00436-022-07697-z 36258094

[19] Jiang X, Wang J. Biological control of Escherichia coli O157: H7 in dairy manure-based compost using competitive exclusion microorganisms. Pathogens. 2024;13(5):361. doi: 10.3390/pathogens13050361 38787213 PMC11124295

[20] Johnson JG, Gaddy JA, DiRita VJ, et al. The PAS domain-containing protein HeuR regulates heme uptake in campylobacter jejuni. MBio. 2016;7(6). doi: 10.1128/mBio.01691-16 PMC5111405 27935836

[21] Ajam-Hosseini M, Akhoondi F, Parvini F, et al. Gram-negative bacterial sRnas encapsulated in OMVs: an emerging class of therapeutic targets in diseases. Front Cell Infect Microbiol. 2023;13:1305510. doi: 10.3389/fcimb.2023.1305510 38983695 PMC11232669

[22] Cernat RC, Scott KP. Evaluation of novel assays to assess the influence of different iron sources on the growth of clostridium difficile. Anaerobe. 2012;18(3):298–304. doi: 10.1016/j.anaerobe.2012.04.007 22554901

[23] Pi H, Sun R, McBride JR, et al. Clostridioides difficile ferrosome organelles combat nutritional immunity. Nature. 2023;623(7989):1009–1016. doi: 10.1038/s41586-023-06719-9 37968387 PMC10822667

[24] Drakesmith H, Zimmermann MB. Another iron in C. difficile’s fire. Cell Host Microbe. 2024;32(1):1–2. doi: 10.1016/j.chom.2023.12.008 38211560

[25] Mridha S, Abt MC. Starvation helps transition to abundance - a ferrosome story. Trends Microbiol. 2024;32(3):219–220. doi: 10.1016/j.tim.2024.01.006 38281864 PMC10967234

[26] Kortman GA, Mulder ML, Richters TJ, et al. Low dietary iron intake restrains the intestinal inflammatory response and pathology of enteric infection by food-borne bacterial pathogens. Eur J Immunol. 2015;45(9):2553–2567. doi: 10.1002/eji.201545642 26046550 PMC4618841

[27] Yang H, Wu X, Li X, et al. A commensal protozoan attenuates clostridioides difficile pathogenesis in mice via arginine-ornithine metabolism and host intestinal immune response. Nat Commun. 2024;15(1):2842. doi: 10.1038/s41467-024-47075-0 38565558 PMC10987486

[28] Shelby RD, Janzow GE, Mashburn-Warren L, et al. A novel probiotic therapeutic in a murine model of clostridioides difficile colitis. Gut Microbes. 2020;12(1):1814119. doi: 10.1080/19490976.2020.1814119 32954922 PMC7524353

[29] Littmann ER, Lee J-J, Denny JE, et al. Host immunity modulates the efficacy of microbiota transplantation for treatment of clostridioides difficile infection. Nat Commun. 2021;12(1). doi: 10.1038/s41467-020-20793-x PMC7854624 33531483

[30] Pereira FC, Wasmund K, Cobankovic I, et al. Rational design of a microbial consortium of mucosal sugar utilizers reduces clostridiodes difficile colonization. Nat Commun. 2020;11(1):5104. doi: 10.1038/s41467-020-18928-1 33037214 PMC7547075

[31] Kelly CR, Yen EF, Grinspan AM, et al. Fecal microbiota transplantation is highly effective in real-world practice: initial results from the FMT National Registry. Gastroenterology. 2021;160(1):183–192.e183. doi: 10.1053/j.gastro.2020.09.038 33011173 PMC8034505

[32] Soutourina O, Dubois T, Monot M, et al. Genome-wide transcription start site mapping and promoter assignments to a Sigma factor in the human Enteropathogen clostridioides difficile. Front Microbiol. 2020;11:1939. doi: 10.3389/fmicb.2020.01939 32903654 PMC7438776

[33] Yang J, Yang H. Transcriptome analysis of the clostridioides difficile response to different doses of bifidobacterium breve. Front Microbiol. 2020;11:1863. doi: 10.3389/fmicb.2020.01863 32849451 PMC7411088

[34] Palmer LD, Skaar EP. Transition metals and virulence in bacteria. Annu Rev Genet. 2016;50(1):67–91. doi: 10.1146/annurev-genet-120215-035146 27617971 PMC5125913

[35] Yang JY, Chen SY, Wu YH, et al. Ameliorative effect of buckwheat polysaccharides on colitis via regulation of the gut microbiota. Int J Biol Macromol. 2023;227:872–883. doi: 10.1016/j.ijbiomac.2022.12.155 36563806

[36] Shine EE, Crawford JM. Molecules from the microbiome. Annu Rev Biochem. 2021;90(1):789–815. doi: 10.1146/annurev-biochem-080320-115307 33770448

[37] Martinez-Gili L, JaK M, Liu Z, et al. Understanding the mechanisms of efficacy of fecal microbiota transplant in treating recurrent clostridioides difficile infection and beyond: the contribution of gut microbial-derived metabolites. Gut Microbes. 2020;12(1):1810531. doi: 10.1080/19490976.2020.1810531 32893721 PMC7524310

[38] Lesniak NA, Schubert AM, Sinani H, et al. Clearance of clostridioides difficile colonization is associated with antibiotic-specific bacterial changes. mSphere. 2021;6(3). doi: 10.1128/mSphere.01238-20 PMC8103992 33952668

[39] Yoon MY, Lee K, Yoon SS. Protective role of gut commensal microbes against intestinal infections. J Microbiol. 2014;52(12):983–989. doi: 10.1007/s12275-014-4655-2 25467115

[40] Robinson JI, Weir WH, Crowley JR, et al. Metabolomic networks connect host-microbiome processes to human clostridioides difficile infections. J Clin Invest. 2019;129(9):3792–3806. doi: 10.1172/JCI126905 31403473 PMC6715368

[41] Reed AD, Fletcher JR, Huang YY, et al. The Stickland reaction precursor trans-4-hydroxy-l-proline differentially impacts the metabolism of clostridioides difficile and commensal clostridia. mSphere. 2022;7(2):e0092621. doi: 10.1128/msphere.00926-21 35350846 PMC9044972

[42] Pike CM, Tam J, Melnyk RA, et al. Tauroursodeoxycholic acid inhibits clostridioides difficile toxin-induced apoptosis. Infect Immun. 2022;90(8):e0015322. doi: 10.1128/iai.00153-22 35862710 PMC9387233

[43] Brandes V, Schelle I, Brinkmann S, et al. Protection from clostridium difficile toxin B-catalysed Rac1/Cdc42 glucosylation by tauroursodeoxycholic acid-induced Rac1/Cdc42 phosphorylation. Biol Chem. 2012;393(1–2):77–84. doi: 10.1515/BC-2011-198 22628301

