# Supplementary figures and images for: Dietary iron attenuates Clostridioides difficile infection via modulation of intestinal immune response and gut microbiota
Source: Virulence. 2025 Jul 16;16(1):2529454. doi: 10.1080/21505594.2025.2529454 (PMC12269695; doi:10.1080/21505594.2025.2529454)

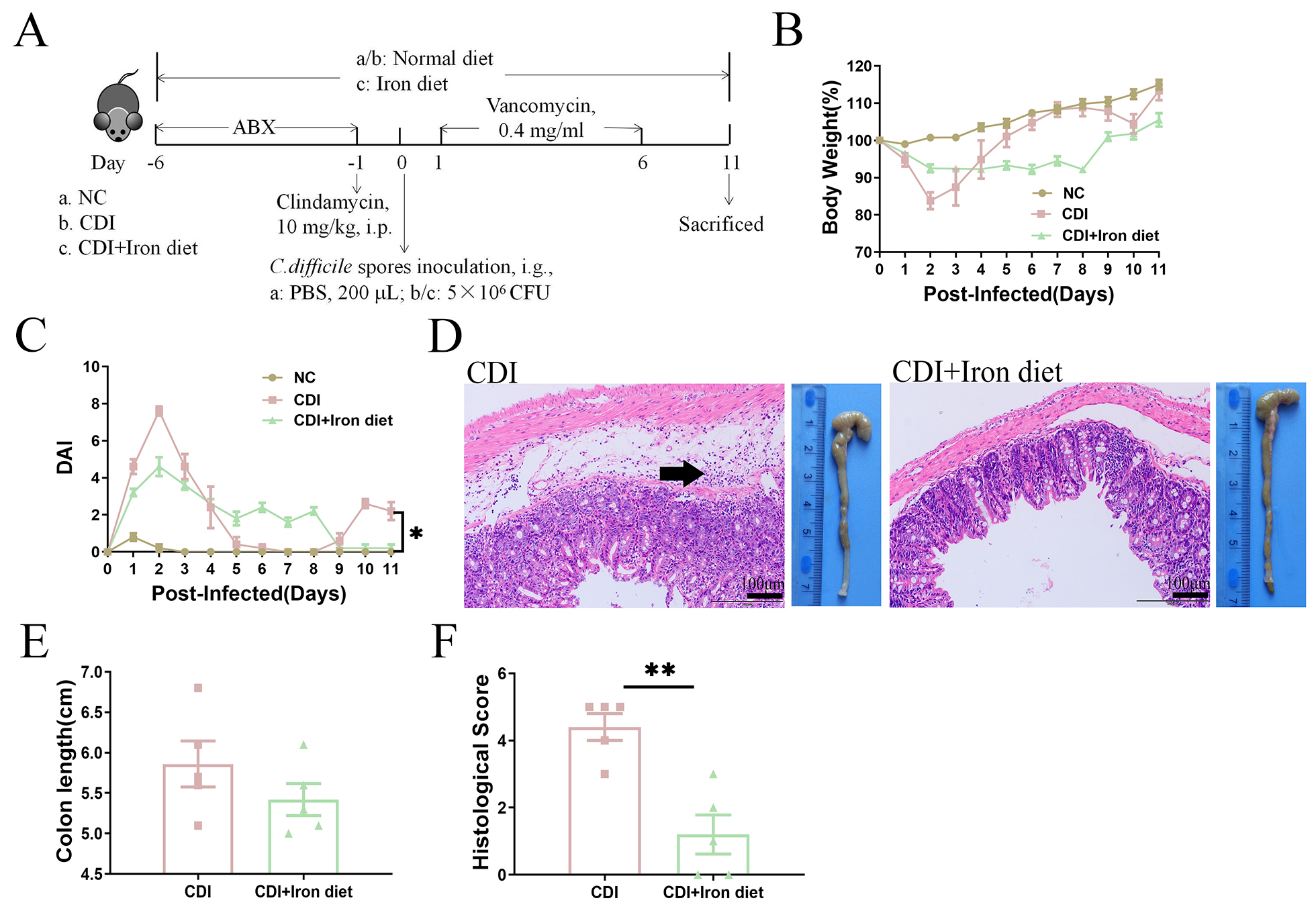

Supplement: Figure S1.jpg [file KVIR_A_2529454_SM1948.jpg]

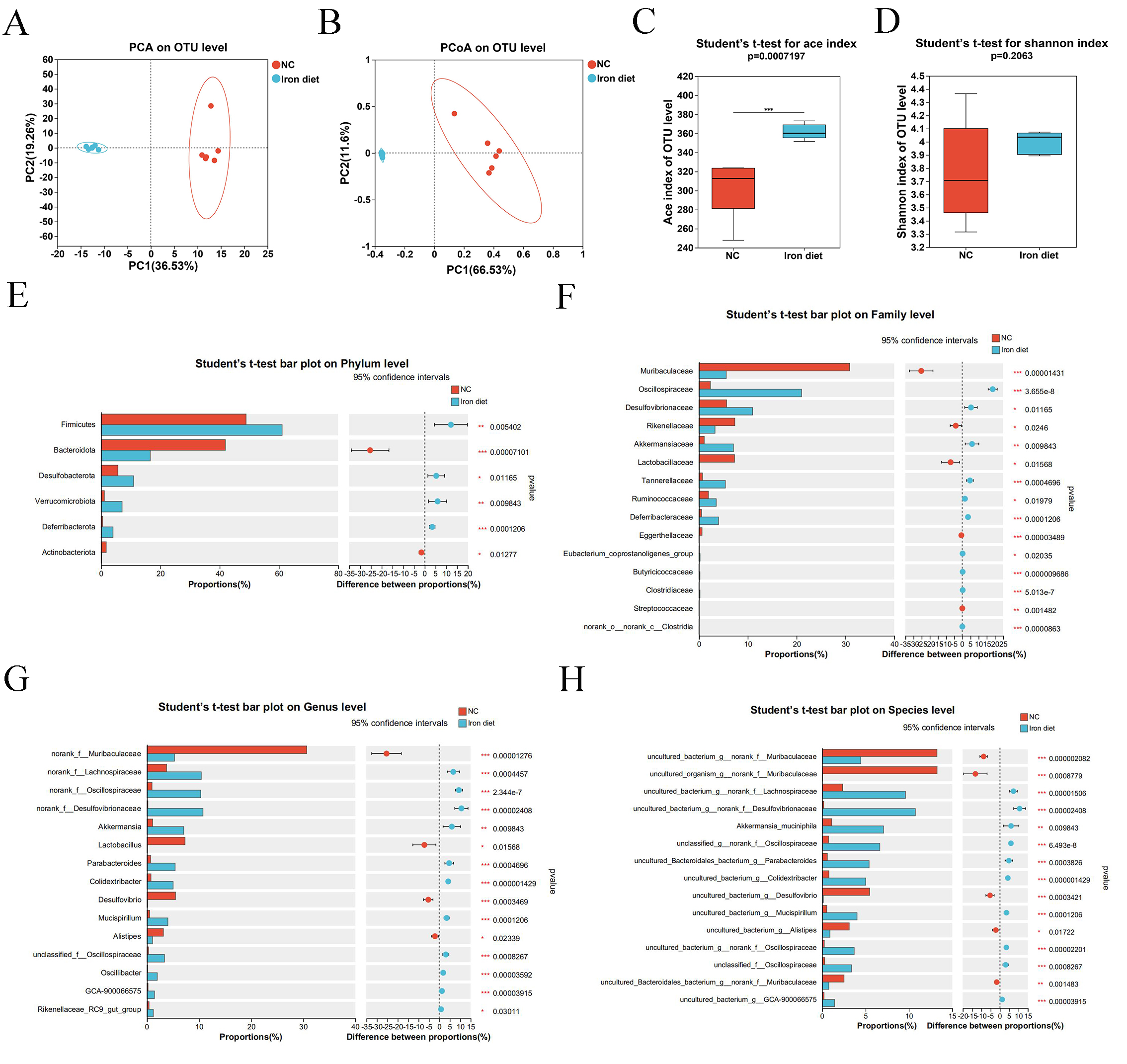

Supplement: Figure S2.jpg [file KVIR_A_2529454_SM1946.jpg]
