# Supplementary material for: Dietary iron attenuates Clostridioides difficile infection via modulation of intestinal immune response and gut microbiota
Source: Virulence. 2025 Jul 16;16(1):2529454. doi: 10.1080/21505594.2025.2529454 (PMC12269695; doi:10.1080/21505594.2025.2529454)
Supplement: Supplementary information.docx [file KVIR_A_2529454_SM1947.docx]

**Supplementary Information**

**Dietary iron attenuates *Clostridioides difficile* infection via modulation of intestinal immune response and gut microbiota**

Xiao Li ^a#^, Xiaoxiao Wu ^a#^, Wanqing Zang ^a#^, Zhou Zhou ^a^, Wenwen Cui ^b^, Ying Chen ^a*^, Huan Yang ^a*^

**Including:** **Supplementary Figures 1-2**

**
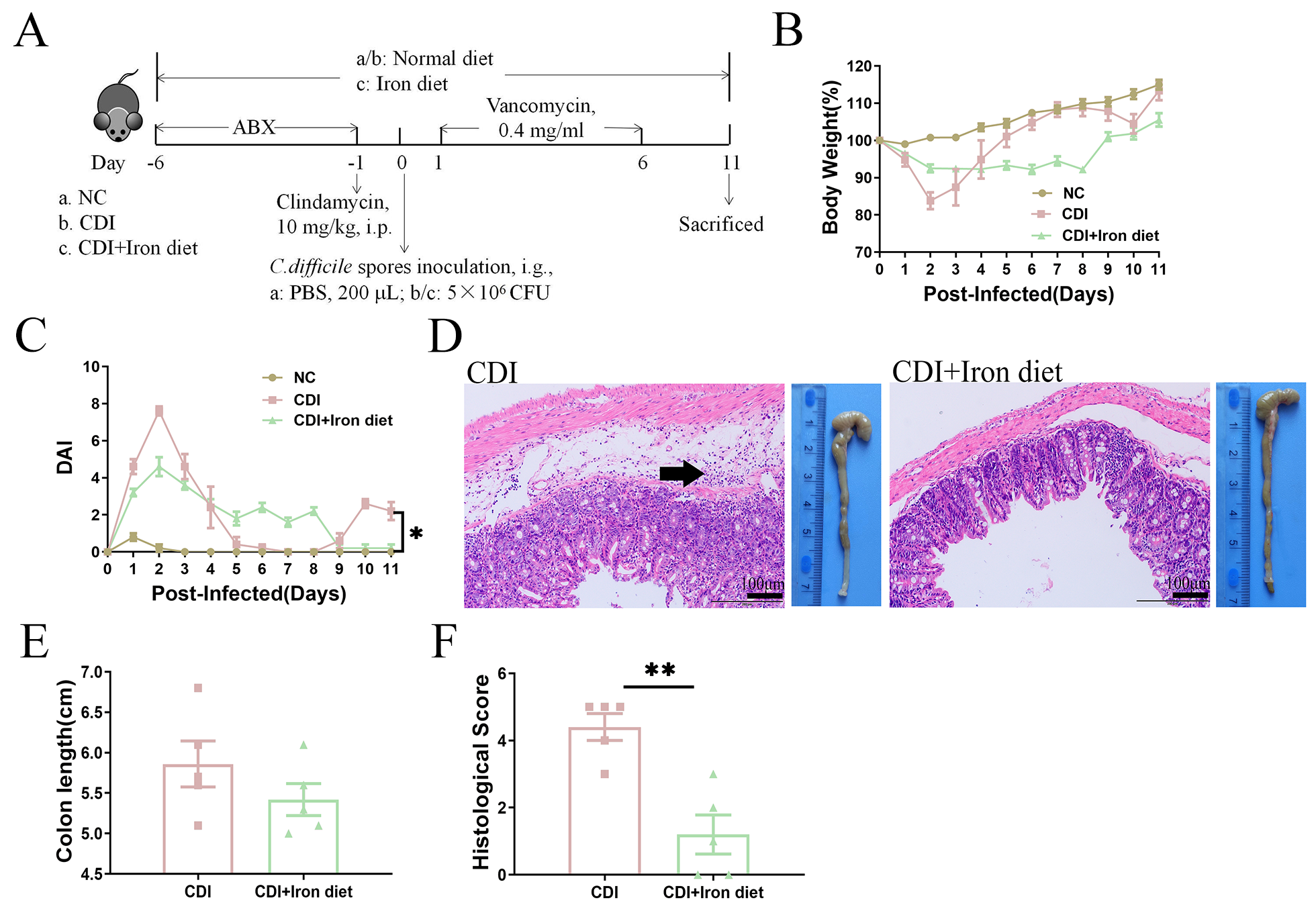
**

**Supplementary Figure 1 | Efficacy of high iron diet against recurrent CDI.** (A) Schematic image illustrating rCDI design. C57BL/6J mice were fed with a normal or high iron (400 mg/kg ferrous sulfate) diet before and throughout the experiment. Mice were treated with ABX for 5 d and then received a single i.p. dose of clindamycin (10 mg/kg). 1 d later, mice were infected with 5×10^6^ CFU of *C. difficile* spores (day 0). On day 1, the mice were given vancomycin (0.4 mg/mL) for 5 d. Cecum and colon were taken from the sacrificed mice after 11 days since infection. (B-C) Mice were monitored for body weight change (B) and DAI (C) every single day (n = 5). (D) Macroscopic photos of colon and representative HE-staining images (200×) of cecum. Scale bar: 100 μm. Arrow indicates the infiltration of inflammatory cells. (E) Measurement and quantification of colon length. (F) Quantitation of histology score in cecum. Data are the mean ± SEM. Statistical significance was determined by two-way ANOVA (B, C) or Mann-Whitney test (E, F), *** p<* 0.01.


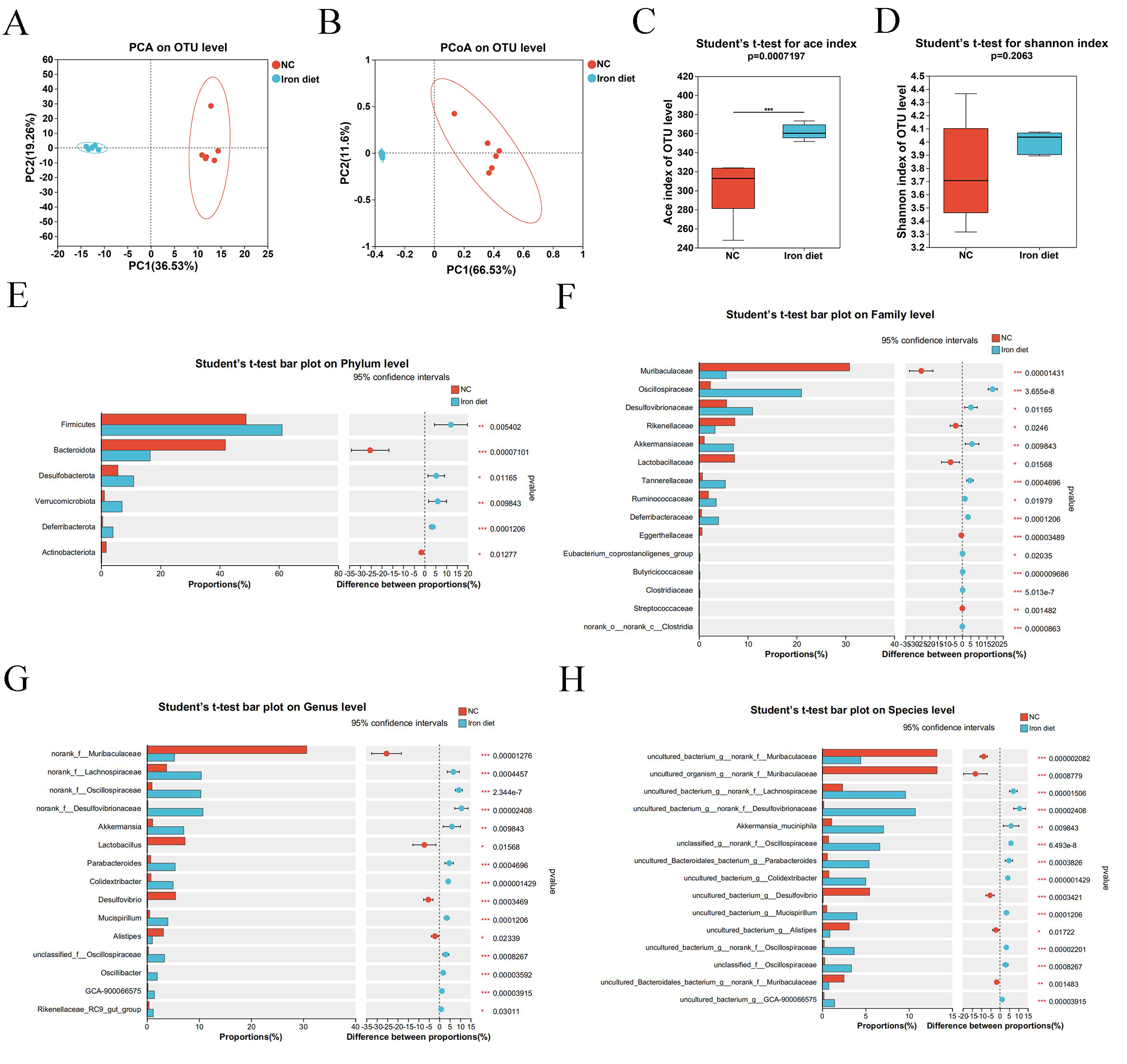


**Supplementary Figure 2 | High iron diet significantly altered the intestinal microbiota.** C57BL/6J mice were fed with the normal or high iron diet for 3 weeks. On day 21, cecal contents from mice fed with normal diet (NC) or high iron diet (Iron diet) were collected and analyzed by 16S rRNA gene sequencing (n = 6). (A) Unweighted principal component analysis (PCA) score plots of OTUs. (B) Unweighted principal coordinate analysis (PCoA) score plots of OTUs. (C) Ace index. (D) Shannon index. (E-H) Multi species comparison bar chart of phylum (E), family (F), genus (G), and species (H). ** p <* 0.05; *** p <* 0.01; **** p <* 0.001.
